# Fluoroquinolone-resistant *Salmonella Typhi* presenting as ileal perforation in a traveler

**DOI:** 10.1093/jscr/rjag557

**Published:** 2026-07-15

**Authors:** Mohammad Aamir Qayyum Sarguroh Khan, Lina Abdel Naser H Hajjar, Gyanendra Jora, Aafreen Ali Nyaz, Mohammad Omran Khalil, Fatema Abdulsalam Alfahim, Muhammad Zubair Khalid, Joaquin Salvelio Picazo Yeste

**Affiliations:** Sheikh Khalifa Medical City, Abu Dhabi 51900, United Arab Emirates; Sheikh Khalifa Medical City, Abu Dhabi 51900, United Arab Emirates; KIST Medical College & Teaching Hospital, Lalitpur, Nepal; NMC Royal Hospital, Khalifa City, Abu Dhabi 35233, United Arab Emirates; Al-Quds University, Jerusalem, Palestine; Sheikh Khalifa Medical City, Abu Dhabi 51900, United Arab Emirates; Sheikh Khalifa Medical City, Abu Dhabi 51900, United Arab Emirates; Sheikh Khalifa Medical City, Abu Dhabi 51900, United Arab Emirates

**Keywords:** typhoid fever, *Salmonella Typhi*, intestinal perforation

## Abstract

Typhoid fever is endemic in South Asia but often overlooked or misdiagnosed in non-endemic countries like the United Arab Emirates. Intestinal perforation is a rare but life-threatening complication, and growing fluoroquinolone resistance has made empirical management more challenging. A 39-year-old male ship worker presented with generalized peritonitis after recent travel to India and had received empirical ciprofloxacin without improvement. Imaging revealed pneumoperitoneum, and laparoscopy identified a single ileal perforation 30 cm proximal to the ileocecal junction, which was repaired. Blood and stool cultures confirmed ciprofloxacin-resistant Salmonella sensitive to trimethoprim-sulfamethoxazole and meropenem. He received 7 days of intravenous meropenem followed by oral Trimethoprim-Sulfamethoxazole, resulting in complete recovery. This case highlights the importance of considering typhoid in travelers with acute abdomen, even in non-endemic regions, and emphasizes culture-guided therapy and reassessment of empirical antibiotics amid rising resistance.

## Introduction

Typhoid fever remains endemic in many parts of South Asia due to poor sanitation [1, 2] but rare in non-endemic countries like the United Arab Emirates (UAE). However, imported infections are increasingly encountered due to global travel [3]. Intestinal perforation is a severe complication of typhoid fever, typically involving the terminal ileum due to necrosis of Peyer’s patches, leading to ulceration and perforation [2, 4]. Despite improvements in both antimicrobial therapy and surgical care, reported death rates remain widely variable, and early recognition is the single most important factor in improving patient outcomes [2, 5]. Clinical presentation is often nonspecific, and delayed diagnosis or inappropriate empirical therapy in the setting of rising antimicrobial resistance increases the risk of complications [1, 6].

Herein, we report a case of typhoid fever presenting in ileal perforation, initially misdiagnosed, emphasizing the importance of early suspicion, travel history awareness, and culture-guided therapy in non-endemic settings.

## Case description

A 39-year-old male presented to the Emergency Department of our tertiary center in Abu Dhabi with severe abdominal pain, vomiting, loose stools, and fever. He appeared acutely ill, dehydrated, and in distress. On arrival, he was febrile (38.4°C), tachycardic (110 bpm), with stable blood pressure (144/76 mmHg). He had been treated at a private clinic 10 days earlier for presumed infective gastroenteritis with ciprofloxacin and diclofenac, without improvement. He had returned from India 28 days prior and worked on a ship. He had no comorbidities. Abdominal examination revealed rigidity, rebound tenderness, and guarding, consistent with generalized peritonitis. Laboratory tests showed white blood cells (WBC) 11.5 × 10^9^/l and C-reactive protein (CRP) 41.6 mg/l. Chest X-ray showed pneumoperitoneum (Fig. 1). Computed tomography (CT) abdomen/pelvis demonstrated a perforated upper gastrointestinal viscus with pneumoperitoneum and free pelvic fluid (Fig. 2), with a possible duodenal wall defect.

**Figure 1:**
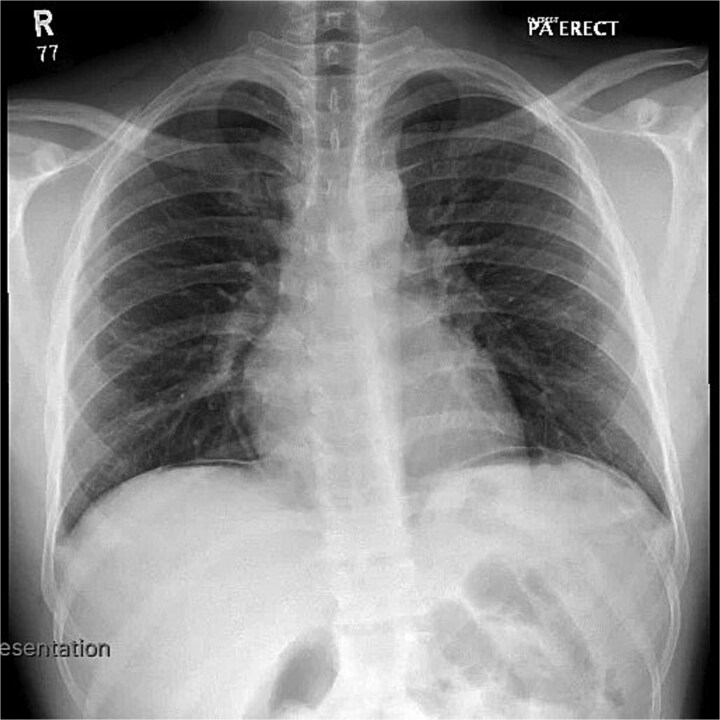
Chest X-ray demonstrating pneumoperitoneum.

**Figure 2:**
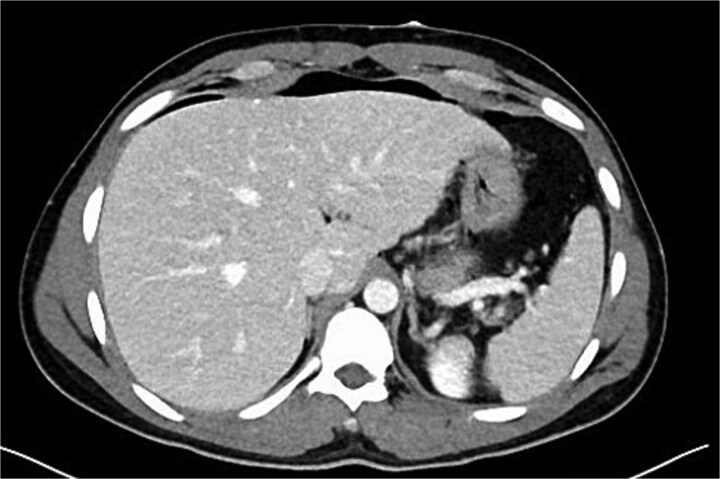
Axial contrast-enhanced CT image of the abdomen showing free intraperitoneal air anterior to the liver (pneumoperitoneum).

Based on the labs and imaging, diagnostic laparoscopy was done for suspected generalized peritonitis due to a perforated viscus. Perioperatively, the peritoneal cavity showed dirty fluid all over the abdomen. The stomach and duodenal area were examined thoroughly, but no perforation was noted. Examination of the distal ileum showed a single perforation (Fig. 3) about 1 foot from the ileocecal junction. It was closed primarily with Polydioxanone 2–0. The entire abdominal cavity was washed with 2 liters of saline. Further, a drain was placed in the pelvis; a stoma was not required in this case. Postoperatively, diagnosis was changed to generalized peritonitis due to possible typhoid perforation in distal ileum.

**Figure 3:**
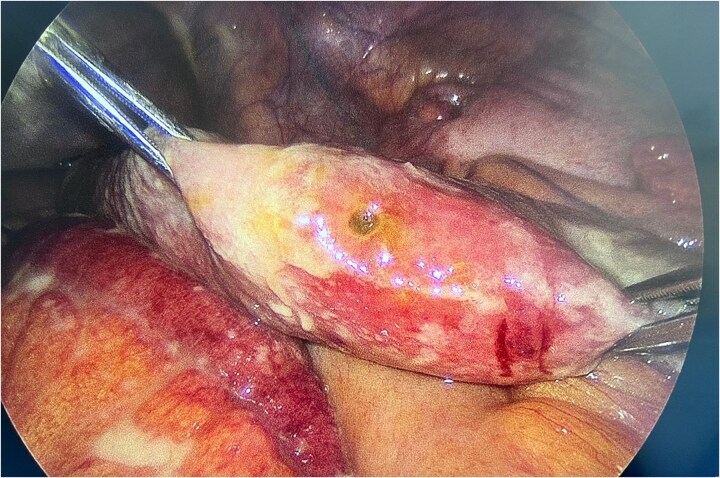
Intraoperative view of the distal ileum demonstrating a single perforation.

Postoperatively, he was managed conservatively with nil per os (NPO), intravenous (IV) fluids, a proton pump inhibitor, analgesia, and IV antibiotics. The drain was 135 ml over 24 hours and gradually decreased over the following days (Table 1). Intraoperatively, due to the presence of ileal perforation, Salmonella infection was suspected. Blood and stool cultures were sent, which later grew Salmonella species, confirming the diagnosis. He was therefore started on IV meropenem for 7 days. Fever gradually subsided, bowel function returned to normal and inflammatory markers trended downward. He gradually transitioned from NPO to a soft diet. Serial examination showed a soft, non-distended abdomen with decreased drain output and removal of drain on postoperative day 7.

**Table 1:** Clinical progression of the patient during hospitalization.

	Admission	Day 1	Day 2	Day 3	Day 4	Day 5	Day 6	Day 7	Day 9 (in clinic)
Temp., maximum (°C)	38.4	36.7	36.8	36.9	36.6	36.8	37.2	36.8	-
Other signs/ symptoms	Nausea, vomiting, loose stools (but no diarrhea), lower abdominal pain for 10 days. Worsening abdominal pain since 1 day. Bilateral lower abdominal tenderness.	Improving abdominal pain.	Distended soft abdomen, improving abdominal pain.	Distended soft abdomen.	Distended soft abdomen, nausea, vomiting.	Nausea, vomiting ×3, diarrhea ×5	Passed stool, no nausea, vomiting, no diarrhea.	Passing watery stools.	Minimal tenderness at drain site.
Oral intake	NPO	Tolerating sips of oral fluid	Clear liquid diet	Soft diet	Full liquid diet	Full liquid diet	Soft diet	Regular diet	Regular diet
Drain output in ml/ 24 hrs		135, dirty fluid	146, serous	140, serous	320, serous	150, serous	16, serous	7, sero-sanguinous Drain removed.	No drain
Antibiotic therapy	Oral ciprofloxacin	IV meropenem						Discharged on Trimethoprim-Sulfamethoxazole for 8 days.	Trimethoprim-Sulfamethoxazole
WBC (10^9^/l)	11.5		12.22	8.7	7.0	6.6			
CRP (mg/l)	41.6		303.5	224		116			
Other labs	Malarial thin/ thick film, parasites antigen negative. Blood culture grew gram-negative rods.	Blood culture (*pre*) grew Salmonella group.	Urine culture shows no growth.	Blood culture (*final*) confirms Salmonella species.	Stool culture (pre) grew Salmonella group.	Stool culture (final) confirms Salmonella species. Stool sent to test for *C. diff* because of diarrhea		*Clostridium difficile* toxin A + B negative.	

His antibiotic sensitivity culture became available (Tables 2 and 3) and it showed resistance to ciprofloxacin and sensitivity to sulfamethoxazole/trimethoprim. Therefore, he was discharged home on cotrimoxazole for 8 days (after 7 days of hospitalization) and was advised to follow up in the clinic. At follow-up, he was afebrile with no abdominal pain or diarrhea. However, he reported anal pain during defecation and was diagnosed with anal fissure, likely secondary to altered bowel habits during recovery. This was managed conservatively with topical treatment and dietary modification.

**Table 2:** Blood culture results with corresponding antibiotic sensitivity patterns.

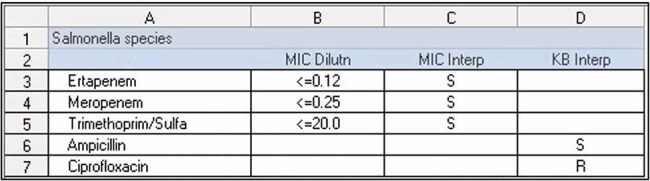

**Table 3:** Stool culture results with corresponding antibiotic sensitivity patterns.

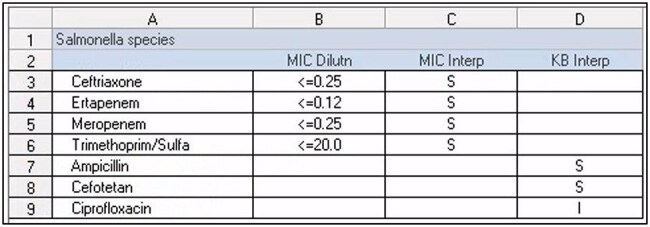

## Discussion

Typhoid fever, caused by *Salmonella enterica serovar Typhi* remains endemic in the Indian subcontinent [8]. Once ingested, the organism invades the intestinal mucosa, survives within macrophages, and disseminates systemically, with a tendency to localize in the Peyer’s patches of the terminal ileum, leading to necrosis, ulceration, and eventual ileal perforation typically along the antimesenteric border [1, 7, 12].

The patient’s recent travel to India and occupation as a ship worker represent significant epidemiological risk factors. The patient’s poor response to ciprofloxacin highlights the growing concern of antimicrobial resistance in Salmonella infections [1]. Thus, treatment was escalated to intravenous meropenem in line with management protocols for severe cases. Upon discharge, antibiotics were de-escalated to oral cotrimoxazole based on sensitivity. This underscores the importance of culture-guided therapy [1].

Intestinal perforation is the most severe complication of typhoid fever, leading to morbidity and mortality [9]. Perforation typically occurs during the second to third week of illness; however, earlier presentations may occur with virulent strains or inappropriate therapy. Reported mortality ranges from 5% to 62% [10, 11]. Outcomes depend more on disease severity, delay in treatment, and patient condition [2]. Surgical management remains the cornerstone of treatment, with options including primary repair, resection with anastomosis, and diversion procedures [2]. Simple primary closure remains the preferred surgical method due to its efficiency and cost-effectiveness [13]. Although exploratory laparotomy has traditionally been the standard approach, diagnostic laparoscopy is increasingly utilized in selected cases. In this patient, laparoscopy successfully identified the ileal perforation, highlighting its diagnostic and therapeutic utility. Moreover, outcomes depend more on early diagnosis, adequate resuscitation, and timely surgical intervention than on the specific operative technique [2].

The differential diagnosis of ileal perforation includes infectious and non-infectious causes. Typhoid fever should be considered in patients with acute abdomen and relevant exposure history [1], even in non-endemic settings such as the UAE, where delayed or missed diagnosis, as observed in this case, may adversely affect outcomes. Blood cultures yield *Salmonella Typhi* in only 3%–34% of typhoid perforation cases, while stool and peritoneal fluid cultures are often negative, further complicating diagnosis [14]. The favorable outcome in this patient can be attributed to prompt surgical intervention, appropriate escalation of antibiotic therapy, and vigilant postoperative care.

This case emphasizes the need to consider typhoid perforation in travelers presenting with acute abdomen, even in non-endemic regions, the critical role of culture-guided therapy amid rising antimicrobial resistance, and that laparoscopic primary repair is safe and effective.
